# Pipe up or clam up: the double-edged sword effect of perceived overqualification on employee voice behavior

**DOI:** 10.3389/fpsyg.2025.1632774

**Published:** 2025-08-18

**Authors:** Shuting Xiang, Dan Xiang, Jiahao Luo, Nuo Chen

**Affiliations:** School of International Business, Southwestern University of Finance and Economics, Chengdu, China

**Keywords:** perceived overqualification, voice behavior, emotional exhaustion, self-efficacy, JD-R model

## Abstract

Existing studies on the relationship between perceived overqualification (POQ) and voice behavior do not reach a consensus. Drawing on the job demands-resources model, this article explored the double-edged sword effect of POQ on voice behavior and its boundary condition. We test our hypotheses based on data collected from 430 employees across a three-wave study. The results demonstrate that POQ is negatively associated with employees’ voice behavior through emotional exhaustion. Conversely, it is also positively associated with voice behavior via self-efficacy. Employees’ careerism orientation careerism orientation moderates the relationship between POQ and self-efficacy, such that the effect is stronger when employees have high levels of careerism orientation.

## 1 Introduction

Statistics shown that nearly half of workers (47%) worldwide perceive themselves as overqualified for their current jobs, particularly those in China (84%), Turkey (78%), and Greece (69%) (Yeşiltaş et al., 2023). Perceived overqualification (POQ) is prevalent in the modern workplace (Li et al., 2024), referring to employees whose educational background, work experience, skills, knowledge, and abilities exceed the requirements of their job positions (Johnson and Johnson, 1996; Maynard and Parfyonova, 2013). It significantly impacts employees’ extra role behavior and overall organizational performance (Liu et al., 2024; Wu et al., 2022; Luksyte et al., 2020; Kim et al., 2019; Chen et al., 2017). Among these behaviors, voice behavior, which is a typical proactive behavior, serves as a catalyst for positive organizational change (Van Dyne et al., 1995) and is considered one of the important factors driving effective organizational functioning (Fu et al., 2023). This has led many scholars to explore the relationship between POQ and voice behavior (Burris, 2012; Wu et al., 2017; Erdogan et al., 2018).

Previous research has yielded mixed findings regarding the effect of POQ on voice behavior. Some scholars argue that POQ is associated with employees feeling undervalued or disrespected, fostering a sense of disengagement and organizational alienation, which inhibits voice behavior (Yang and Li, 2021; Wu et al., 2017). Whereas POQ can enhance employees’ positive self-concept and problem-solving abilities, stimulating their motivation to voice opinions, thereby exerting a positive influence on voice behavior (Erdogan et al., 2018; Zhou et al., 2020). These contrasting findings suggest that POQ may not exert a unidimensional effect, but rather function as a double-edged sword, simultaneously triggering both promotive and inhibitive forces on employee voice behavior. Although prior studies have separately documented the positive and negative outcomes of POQ, an integrated theoretical model explaining how and why POQ simultaneously stimulates and suppresses voice behavior remains lacking. This study seeks to address this research gap by employing the job demands-resources (JD-R) model to examine the dual influence pathways of POQ on voice behavior and the boundary conditions.

According to the JD-R, job characteristics are categorized into job demands and job resources, which influence employee behavior via health-impairment and motivational pathways, respectively (Demerouti et al., 2001; Schaufeli and Bakker, 2004). POQ, as a dual-faceted job perception, encompasses both job demand and job resource components. On one hand, it reflects the psychological strain caused by a mismatch between an individual’s capabilities and job requirements, representing a demand attribute that may relate to emotional exhaustion and suppress voice behavior. On the other hand, it also signifies the possession of surplus knowledge and skills beyond job needs, representing a resource attribute that can enhance self-efficacy and promote voice behavior. Furthermore, the JD-R model emphasizes that personal characteristics play a crucial role in shaping how job demands and resources influence psychological outcomes (Bakker and Demerouti, 2017). In line with this, the present study introduces careerism orientation as a key individual difference variable to moderate the psychological effects of POQ. Careerism orientation reflects an individual’s instrumental motivation to pursue personal advancement and career development (Adams et al., 2013), which may increase individuals’ sensitivity to the psychological effects of POQ.

This study makes three key contributions to the literature. First, it introduces a double-edged sword perspective to understand the impact of POQ on employee voice behavior. While prior studies have found both negative (e.g., Yang and Li, 2021; Wu et al., 2017) and positive effects (e.g., Erdogan et al., 2018; Zhou et al., 2020), few have systematically integrated these opposing outcomes within a unified framework. Second, drawing on the JD-R model (Demerouti et al., 2001; Schaufeli and Bakker, 2004), the study develops a dual-pathway model that identifies emotional exhaustion and self-efficacy as parallel mechanisms. This advances POQ research by explaining how it can simultaneously deplete and motivate. Third, the study introduces careerism orientation as a personal value that shapes individual responses to POQ. By highlighting its moderating role, the study extends prior work on boundary conditions of POQ.

## 2 Theoretical review and research hypotheses

### 2.1 Job demands-resources model

The JD-R model, proposed by Demerouti et al. (2001), categorizes all job characteristics into two broad domains: job demands and job resources. Job demands refer to physical, psychological, social, or organizational aspects of work that require sustained effort and are typically associated with physiological or psychological costs, such as workload, time pressure, role conflict, and emotional labor. Job resources, in contrast, encompass aspects of the job that help achieve work goals, reduce job demands and their associated costs, or promote personal development such as feedback, autonomy, organizational support, and training opportunities (Bakker and Demerouti, 2007). The core mechanism of the JD-R model lies in its dual-path hypothesis, which posits that job demands primarily trigger a health-impairment process contribute to strain and reduced work performance, whereas job resources activate a motivational process that enhances intrinsic motivation and proactive work behavior (Demerouti et al., 2001; Bakker and Demerouti, 2017). Furthermore, personal resources such as self-efficacy and optimism function similarly to job resources by bolstering motivation and buffering the negative impact of high job demands (Xanthopoulou et al., 2009; Bakker and Demerouti, 2017).

Drawing on the JD-R model, this study proposes a framework to explain how POQ affects employees’ voice behavior. POQ, as a demand-related stressor involving role underutilization or value misalignment, POQ may induce emotional exhaustion and inhibit voice through the health-impairment process. Conversely, as a subjective job characteristic, reflects employees’ perception that their qualifications exceed job demands. It exhibits a dual nature: as a resource, POQ can enhance self-efficacy by strengthening employees’ confidence and control, thus activating the motivational pathway and encouraging voice. Moreover, the JD-R emphasizes that personal characteristics play a crucial role. Accordingly, this study incorporates careerism orientation as a representative personal trait to examine its moderating effect. Careerism orientation reflects employees’ motivation to pursue status, influence, and upward mobility within the organization (Adams et al., 2013; Yang et al., 2016). Under conditions of POQ, individuals with high careerism are more likely to interpret their surplus qualifications as resources for advancement, thereby strengthening the positive impact of POQ on self-efficacy. Conversely, when their abilities are overlooked, they may experience heightened frustration and strain, reinforcing the emotional costs of POQ.

**FIGURE 1:**
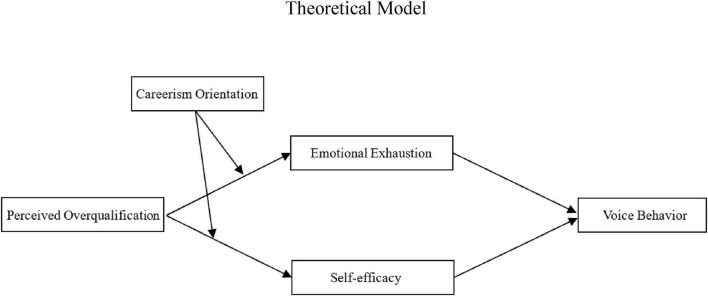
Theoretical model.

In summary, guided by the JD-R model, this study integrates the motivational and health-impairment pathways and considers individual differences to elucidate how POQ influences employee voice behavior (Figure 1).

### 2.2 Perceived overqualification and voice behavior

Employee voice behavior is regarded as a proactive behavior and a form of organizational citizenship behavior. Van Dyne et al. (2003) defines it as “constructive communication suggestions aimed at improving organizational conditions,” categorizing it into promotive voice and prohibitive voice. Building on this framework, Song et al. (2020) further classify voice behavior based on direction into three types: peer-to-peer, upward (subordinate to superior), and downward (superior to subordinate). Among these, voice upward is the most commonly studied, referring to employees’ efforts to make suggestions or express concerns aimed at improving organizational operations (Song et al., 2020). This article will primarily focus on employees’ voice behavior directed toward their superiors or the organization.

Employees experiencing POQ are more inclined to leverage their surplus resources to provide suggestions for the organization, aiming to acquire new resources and subsequently alter their circumstances (Chen and Zhou, 2025; Duan et al., 2022; Grant, 2013). Specifically, first, employees with POQ possess knowledge, experience, and skills that exceed job requirements (Liu et al., 2025; Maynard and Parfyonova, 2013), granting them relatively abundant job resources. Voice behavior can help individual manage their surplus resources (Ng and Feldman, 2012), which may encourage POQ employees to invest more resources in proposing creative, novel, and useful ideas and suggestions. Second, POQ individuals are perceived as capable by team members due to their extensive knowledge and skills, positioning them centrally within the team. This centrality enables them to access various resources more easily, making them more willing to engage in voice behavior to acquire additional resources (Qin et al., 2014). Consequently, their frequency of voice behavior is higher than that of employees in marginal positions (Venkataramani et al., 2016). Third, employees with POQ have the capability to handle excess work tasks (Erdogan et al., 2011; Russell et al., 2016) and tend to demonstrate this ability through positive behaviors such as innovation and proactivity (Luksyte and Spitzmueller, 2016; Deng et al., 2018). Fourth, employees with POQ have a strong desire for challenging work, as their current roles do not fully utilize their capabilities (Simon et al., 2019). To enhance work challenges, they may choose to engage in voice behavior to improve their current work situation, with their organizational citizenship behavior contributing to alleviating unfavorable circumstances (Liu and Wang, 2012; Lin et al., 2017). Erdogan et al. (2018) find that employees experiencing POQ are more willing to engage in voice behavior. Based on this, the following hypothesis is proposed:

*H1: Perceived overqualification is positively related to employees’ voice behavior.*

### 2.3 The mediating role of emotional exhaustion

Emotional exhaustion is a core dimension of job burnout, defined as a state of fatigue resulting from the excessive use and depletion of an individual’s emotional and psychological resources (Maslach et al., 2001). It manifests as a complete loss of interest in a particular matter and serves as a response to stressors in the workplace.

The JD-R model suggests that job demands reduce individual work engagement and increase work-related stress (Demerouti et al., 2001; Schaufeli and Bakker, 2004; Bakker and Demerouti, 2007; McGonagle et al., 2015). Employees experiencing POQ feel that their knowledge and skills exceed job requirements, which is associated with a mismatch and a sense of job inadequacy (Liu et al., 2025; Maynard and Parfyonova, 2013). This perception heightens their feelings of job insecurity (Peiró et al., 2012), a typical job demand that increases stress and depletes psychological or physiological resources. As stress accumulates and resources dwindle, individuals are at a higher risk of experiencing emotional exhaustion (Leiter and Maslach, 1988). Moreover, existing research has verified that POQ significantly affects individuals’ negative emotions. For instance, studies have shown that POQ may contribute to dissatisfaction (Erdogan et al., 2011), anger, low self-esteem (Liu et al., 2015), and feelings of boredom (Watt and Hargis, 2010). Accompanied by this series of negative emotional experiences, individuals with POQ often feel an escalation of work-related stress (Motowidlo et al., 1986), with excessive pressure potentially contributing to emotional exhaustion (Pines and Maslach, 1978; Wang and Zhang, 2016). Therefore, this article posits that POQ can result in individuals falling into a state of emotional exhaustion.

According to the JD-R (Demerouti et al., 2001; Schaufeli and Bakker, 2004; Bakker and Demerouti, 2017), when employees are exposed to persistently high job demands while lacking sufficient resources, they are likely to enter the health-impairment process, which is characterized by the depletion of energetic resources and passive coping behaviors. Emotional exhaustion, as a core outcome of this process, reflects the depletion of emotional and cognitive resources, typically manifested in physical fatigue, cognitive sluggishness, and emotional irritability (Maslach et al., 2001), which in turn suppresses employees’ propensity to engage in voice behavior. Specifically, emotionally exhausted employees often experience dual depletion of physical and psychological energy, accompanied by negative affective responses such as frustration and irritability (Chen and Qin, 2011; Tang et al., 2025), which undermines their level of work engagement (Lin et al., 2020) and reduces the likelihood of initiating spontaneous and proactive behaviors (Bakker et al., 2014). Voice behavior, as a high-level and informal proactive behavior, requires the allocation of additional time, energy, and cognitive resources (LePine and Van Dyne, 1998; Fu et al., 2023). When employees perceive themselves to be in a state of exhaustion, they are more inclined to activate resource conservation mechanisms (Hobfoll, 1989) to prevent further resource loss, thereby reducing their engagement in voice behaviors. Furthermore, insufficient resources not only constrain individuals’ energetic mobilization but also impair the motivational expression of work behaviors. Employees experiencing high levels of emotional exhaustion may demonstrate impaired cognitive functioning, diminished problem-solving abilities, and weakened organizational commitment (Crawford et al., 2010; Tang et al., 2025), which collectively hinder their capacity to make constructive contributions. Emotional exhaustion may also contribute to affective detachment, further eroding employees’ sense of organizational identification and responsibility (Ozcelik, 2017), and suppressing their willingness to offer constructive suggestions. Supporting this perspective, empirical findings by Lin and Johnson (2014) indicate that higher levels of emotional exhaustion significantly predict a subsequent decline in employees’ voice behaviors. Based on this discussion, the following hypothesis is proposed:

*H2: Emotional exhaustion mediates the relationship between perceived overqualification and employees’ voice behavior.*

### 2.4 The mediating role of self-efficacy

Self-efficacy refers to a cognitive state in which an individual predicts their ability to complete a specific task or job, reflecting their belief or confidence in their capabilities (Bandura, 1986). Based on the JD-R (Demerouti et al., 2001; Schaufeli and Bakker, 2004), POQ reflects employees’ perception of surplus resources and abilities, which can be regarded as a motivational job resource. This subjective resource enhances employees’ confidence in managing their work environment, thereby fostering higher levels of self-efficacy. As a key personal resource, self-efficacy not only stimulates employees’ intrinsic work motivation but also increases their willingness and capability to engage proactively (Bakker and Demerouti, 2017), enabling them to more actively identify and address problems within the work environment, which significantly promotes the occurrence of voice behavior.

Research indicates that the development of self-efficacy involves three types of evaluative analyses: analysis of task requirements, attributional analysis of experience, and assessment of personal and situational resources (Gist and Mitchell, 1992; Den Hartog and Belschak, 2012). First, when analyzing task requirements, individuals with POQ possess abundant abilities, skills, and experiences, which enable them to assess and handle work tasks more proficiently (Lee et al., 2021), resulting in a higher sense of self-efficacy. Second, in the process of attributional analysis of experience, POQ individuals demonstrate stronger capabilities and resources, allowing them to excel in task completion. This success garners recognition and encouragement from others, fostering self-attribution and promoting the formation of enhanced self-efficacy. Lastly, in the assessment of personal and situational resources, POQ individuals benefit from abundant job resources (Maynard et al., 2006; Li et al., 2019), providing them with effective support and fewer constraints, which may foster a more favorable self-assessment of their abilities. Consequently, this article posits that individuals with POQ perceive their skills as exceeding job requirements, making them more willing to utilize their talents to undertake additional tasks. This reflects their judgment and confidence in their ability to complete and execute the tasks of their current roles (Tierney and Farmer, 2011), thereby fostering a higher sense of self-efficacy.

According to the JD-R model (Demerouti et al., 2001; Schaufeli and Bakker, 2004; Bakker and Demerouti, 2017), personal resources such as self-efficacy function similarly to job resources within the work environment (Bakker and Demerouti, 2017), significantly enhancing individuals’ intrinsic motivation and work engagement (Gonzalez-Mulé et al., 2021). Self-efficacy, as a key cognitive resource, not only increases employees’ perceived control over their work context (Bakker and Schaufeli, 2008) but also fosters positive cognitive and affective states, thereby boosting work passion and effort investment (Bakker and Demerouti, 2007; Xanthopoulou et al., 2009). This positive state enables employees to better utilize their knowledge and skills, proactively generating innovative and constructive suggestions (Zhang et al., 2016), which constitute a fundamental psychological basis for voice behavior. Moreover, individuals with elevated self-efficacy demonstrate stronger work motivation, actively acquiring task-relevant information, which increases their job involvement and engagement, thereby eliciting greater proactive behaviors (Ma et al., 2023). This reciprocal cognitive-behavioral dynamic facilitates the ideation, dissemination, and implementation of novel concepts (Ng and Lucianetti, 2016). Empirical findings corroborate that self-efficacy serves as a significant positive predictor of voice behavior (Walumbwa et al., 2010). Based on the above discussion, the following hypothesis is proposed:

*H3: Self-efficacy mediates the relationship between perceived overqualification and employees’ voice behavior.*

### 2.5 The moderating role of careerism orientation

Careerism orientation refers to an employee’s tendency to pursue career development through non-performance-related means (Feldman and Weitz, 1991). It reflects an individual’s career values and attitudes regarding self-determination, promotion, mobility, organizational support, and security, driving career choices (Tschopp et al., 2014). This orientation, embodying a motivational tendency toward rapid career advancement, aligns with the fundamental characteristics of personal resources (Bakker and Demerouti, 2017). On the one hand, it may heighten sensitivity to resource wastage, thereby influencing the activation of stress pathways; on the other hand, it can stimulate individuals to identify and utilize work-related resources proactively. Thus, within the JD-R theoretical framework, a careerism orientation functions not only as a stable individual trait but also as a resource investment strategy that shapes employees’ cognitive appraisals and coping mechanisms when facing a qualification surplus.

The health-impairment process in the JD-R model emphasizes how individuals perceive and respond to stress induced by job demands (Demerouti et al., 2001; Bakker and Demerouti, 2017). Employees with high careerism orientation typically regard work as a conduit to achieve career goals and highly value maximizing resource utility (Tschopp et al., 2014; Yang et al., 2016). When they feel their abilities are underestimated or lack development opportunities, they tend to experience intense frustration and feelings of suppression (Yeşiltaş et al., 2023). This cognitive imbalance may exacerbate their subjective perception of resource wastage, accelerating resource depletion and contributing to higher levels of emotional exhaustion. In other words, the stronger the careerism orientation, the lower the tolerance for “resource wastage” and the more sensitive the negative perception of qualification surplus, resulting in a more intense stress response within the health-impairment pathway. Conversely, employees with low careerism orientation tend to have weaker expectations for promotion opportunities. They may tolerate mismatches between position and ability to a greater extent, exhibiting a relatively milder resource depletion response. Therefore, the following hypothesis is proposed:

*H4a: Careerism orientation positively moderates the relationship between perceived overqualification and emotional exhaustion, such that the positive relationship between perceived overqualification and emotional exhaustion is stronger for employees with high level of careerism orientation.*

In the JD-R model, how individuals respond to job resources largely depends on their intrinsic motivational tendencies (Demerouti et al., 2001; Bakker and Demerouti, 2017). Careerism orientation reflects employees’ psychological tendency to prioritize career advancement as a core goal (Adams et al., 2013; Yang et al., 2016). This orientation may motivate employees to proactively leverage their surplus capabilities in constructing career development paths. Therefore, employees with higher careerism orientation are more likely to perceive qualification surplus as a strategic resource that can be used to achieve career goals and are more inclined to actively transform it into confidence and competence in their jobs, thereby boosting their self-efficacy (Bandura, 1997). In contrast, employees with lower careerism orientation may lack the intrinsic motivation to convert surplus capabilities into positive resources, resulting in less proactive utilization of qualification surplus and minimal improvement in self-efficacy. Accordingly, it is inferred that careerism orientation positively moderates the relationship between perceived qualification surplus and self-efficacy, making this pathway more significant at higher levels of careerism orientation. Therefore, the following hypothesis is proposed:

*H4b: Careerism orientation positively moderates the relationship between perceived overqualification and self-efficacy, such that the positive relationship between perceived overqualification and self-efficacy is stronger for employees with high level of careerism orientation.*

### 2.6 The moderated mediation model

The previous discussion on the mediating roles of emotional exhaustion and self-efficacy aims to reveal the psychological mechanisms and internal logic behind the double-edged sword effect of POQ on employee voice behavior. Additionally, the exploration of the moderating role of careerism orientation seeks to clarify the conditions under which POQ has a stronger impact on emotional exhaustion and self-efficacy, specifically in scenarios characterized by high levels of careerism orientation. To comprehensively examine the effects and scope of careerism orientation within the theoretical model proposed in this article, a moderated mediation model is further developed based on hypotheses H2, H3, H4a, and H4b.

The emotional pathway, as described, indicates that emotional exhaustion mediates the relationship between POQ and employee voice behavior. Employees with POQ face higher job demands that significantly deplete their psychological and physiological resources, and often experience emotional exhaustion. This sense of burnout triggers their resource protection mechanisms, consequently reducing their voice behavior. Additionally, careerism orientation positively moderates the relationship between POQ and emotional exhaustion. Individuals with high careerism orientation place great emphasis on maximizing the value of resources (Rousseau, 1990; Yang et al., 2016). When they perceive their qualifications as being wasted or their career aspirations unfulfilled, this mismatch may be experienced as a job demand, leading to resource depletion and intensified emotional exhaustion, which in turn reduces their propensity to engage in voice behavior. Therefore, the indirect effect of POQ on employee voice behavior through emotional exhaustion is further intensified by the level of careerism orientation. Consequently, the following hypothesis is proposed:

*H5a: Careerism orientation moderates the indirect effect of perceived overqualification on employee voice behavior through emotional exhaustion. That is, the indirect effect is stronger when POQ employee employees have high levels of careerism orientation.*

The cognitive pathway, as previously discussed, indicates that self-efficacy mediates the relationship between POQ and employee voice behavior. Employees with POQ possess surplus knowledge, skills, and experience, enhancing their perception of individual capability and positively influencing their self-efficacy. This cognitive resource tends to enhance work engagement, ultimately stimulating voice behavior. Additionally, careerism orientation positively moderates the relationship between POQ and self-efficacy. Individuals with high careerism orientation tend to transform surplus qualifications into valuable work resources to advance their career development, thereby enhancing their self-efficacy. This process motivates them to proactively utilize work resources and increases their likelihood of engaging in voice behavior. Consequently, the indirect effect of POQ on employee voice behavior through self-efficacy is further intensified by the level of careerism orientation. Thus, the following hypothesis is proposed:

*H5b: Careerism orientation moderates the indirect effect of perceived overqualification on employee voice behavior through self-efficacy. That is, the indirect effect is stronger when POQ employees have high levels of careerism orientation.*

## 3 Materials and methods

### 3.1 Participants

A three-wave longitudinal study was conducted among 800 frontline employees of a large multinational financial enterprise in southwest China. The respondents were completely anonymous in the process of filling in the questionnaires and obtained certain material rewards upon completion. At Time 1 (T1), 800 employees were invited to participate in the study (response rate 66.5%; *N* = 532) to complete the questionnaire about control variables, POQ and careerism orientation. One month later (T2), the same employees were invited to participate again (response rate 68.5%; *N* = 548) to measure emotional exhaustion and self-efficacy. After another 1 month (T3), the same employees (response rate 63.5%; *N* = 508) were invited to obtain data about voice behavior. After matching data and sorting out invalid questionnaires, 53.8% of the initial samples (*N* = 430) was included in our empirical analysis.

### 3.2 Measures

All measures used have been validated in previous research. Given that all administered items were in Chinese, translation and back-translation procedures were followed to ensure the quality of translations (Brislin, 1986). Each measure used a 6-point Likert-type scale ranging from “strongly disagree” to “strongly agree.”

#### 3.2.1 Perceived overqualification (T1)

Perceived overqualification was measured with Erdogan and Bauer’s (2009) four-item scale (Cronbach α = 0.87), which refined from Johnson and Johnson (1996). Example item: “My formal education overqualifies me for my present job.”

#### 3.2.2 Emotional exhaustion (T2)

For emotional exhaustion, the three-item scale was adapted from Watkins et al.’s (2014) measure (Cronbach α = 0.91). Example item: “I feel emotionally drained from my work.”

#### 3.2.3 Self-efficacy (T2)

To assess self-efficacy, we used Chen et al.’s (2001) eight-item scale (Cronbach α = 0.95). Example item: “I will be able to achieve most of the goals that I have set for myself.”

#### 3.2.4 Voice behavior (T3)

Voice behavior, as a sub-dimension of organizational citizenship behavior. We used the three-item subscale (Cronbach α = 0.86) for voice from Fox et al.’s (2007) organizational citizenship behavior ten-item scale. Example item: “Actively raises suggestions to improve work procedures or process.”

#### 3.2.5 Careerism orientation (T1)

Careerism orientation was measured with Robinson and Rousseau’s (1994) five-item scale (Cronbach α = 0.71). Example item: “I took this job as a stepping stone to a better job with another organization.”

#### 3.2.6 Control variables (T1)

In reference to existing related research (Duan et al., 2022; Li et al., 2024), we controlled several demographic characteristics of employees: gender, age, education level, and tenure.

## 4 Results

### 4.1 Confirmatory factor analysis

We conducted confirmatory factor analysis via Mplus 7.4 to assess the discriminant validity of the measurement model. The results in Table 1 indicated that the hypothesized five-factor model fits the data well (χ^2^/df = 2.34, RMSEA = 0.06, SRMR = 0.06, CFI = 0.96, and TLI = 0.95). The results provided support for taking the fiver constructs as distinctive variables, and the five-factor model was thus retained for substantial hypothesis tests.

**TABLE 1:** Confirmatory factor analysis model fit results.

Models	χ^2^/df	RMSEA	SRMR	CFI	TLI
***Five-factor model***					
The hypothesized five-factor model	2.34	0.06	0.06	0.96	0.95
***Four-factor model***					
Combining POQ and voice behavior	6.97	0.12	0.11	0.80	0.77
Combining POQ and self-efficacy	8.31	0.13	0.13	0.75	0.72
***Three-factor model***					
Combining POQ, careerism orientation and voice behavior	8.42	0.13	0.12	0.74	0.71
Combining POQ, emotional exhaustion and voice behavior	10.77	0.15	0.13	0.66	0.62
***Two-factor model***					
Combining POQ, emotional exhaustion, careerism orientation, and voice behavior	12.10	0.16	0.14	0.61	0.57
Combining POQ, self-efficacy, careerism orientation, and voice behavior	12.60	0.16	0.15	0.59	0.55
***One-factor model***					
Combining all variables	16.42	0.19	0.17	0.46	0.40

*N* = 430. POQ, perceived overqualification; χ^2^, Chi-square; df, degrees of freedom; RMSEA, root mean square error of approximation; CFI, comparative fit index; TLI, Tucker-Lewis index.

### 4.2 Test of common method bias

Due to all data were collected from single source, the potential impacts of common method bias should be examined. As shown in Table 1, the hypothesized five-factor model (χ^2^/df = 2.34, RMSEA = 0.06, SRMR = 0.06, CFI = 0.96, and TLI = 0.95) demonstrates better model fit indexes than the one-factor model (χ^2^/df = 16.42, RMSEA = 0.19, SRMR = 0.17, CFI = 0.46, and TLI = 0.40). In addition, we applied the single unmeasured latent method. The results indicate that the model fit did not significantly change after incorporating the latent method factor into the five-factor model (ΔRMSEA = 0.00, ΔSRMR = 0.00, ΔCFI = 0.00, and ΔTLI = 0.00). Thus, common method bias was not a severe problem in this study.

### 4.3 Multicollinearity diagnostics

Before conducting regression analyses, we tested for multicollinearity among the predictor variables. We calculated variance inflation factors (VIFs), tolerance index (TI), and condition index (CI). As shown in the Table 2, all VIF values were below 2, all TIs exceeded 0.8, and the maximum CI was 22.22 below the critical threshold of 30. These results suggest that multicollinearity was not a serious concern.

**TABLE 2:** Multicollinearity diagnostics.

Predictor	VIF	Tolerance	Condition index
1. Perceived overqualification	1.21	0.83	9.89
2. Emotional exhaustion	1.07	0.94	13.29
3. Self-efficacy	1.02	0.98	22.22
4. Careerism orientation	1.21	0.82	11.37

### 4.4 Hypothesis test

Table 3 provides the means, SDs, correlations, and reliabilities of all the variables in this study. The reliability was above 0.80 for all variables except for careerism orientation. As expected, POQ was significantly correlated with emotional exhaustion (*r* = 0.18, *p* < 0.01), and emotional exhaustion was significantly correlated with voice behavior (*r* = −0.12, *p* < 0.05). POQ was significantly correlated with self-efficacy (*r* = 0.13, *p* < 0.01), and self-efficacy was significantly correlated with voice behavior (*r* = 0.29, *p* < 0.01).

**TABLE 3:** Means, standard deviations, correlations, and reliabilities of studied variables.

Variable	Mean	SD	1	2	3	4	5	6	7	8	9
1. Gender	1.69	0.46	—								
2. Age	3.50	0.25	0.17**	—							
3. Education	2.81	0.60	−0.15**	−0.55**	—						
4. Tenure	3.10	1.67	0.10	0.38**	−0.24**						
5. Perceived overqualification	3.74	0.85	−0.15**	−0.11*	0.19**	−0.05	(0.87)				
6. Emotional exhaustion	3.80	0.98	0.03	−0.07	−0.01	0.09	0.18**	(0.91)			
7. Self-efficacy	4.06	0.66	−0.09	−0.07	0.07	−0.05	0.13**	0.01	(0.95)		
8. Voice behavior	3.91	0.73	−0.07	−0.00	0.08	−0.00	0.13**	−0.12*	0.29**	(0.86)	
9. Careerism orientation	3.65	0.75	−0.08	−0.13**	0.15**	−0.07	0.38**	0.23**	−0.01	0.07	(0.71)

*N* = 430. Cronbach’s alphas are shown in parentheses along the diagonal. SD, standard deviation. Gender: 1 = *male*, 2 = *female*; education level: 1 = *high school or below*, 2 = *junior college*, 3 = *undergraduate*, 4 = *master*, 5 = *doctor*; age and tenure were log-transformed using natural logarithms. *p < 0.05, **p < 0.01.

Hypothesis development was conducted in Mplus 7.4. All control variables (i.e., age, gender, education, and tenure) were included. The results are presented in Tables 4, 5. Results showed that POQ was positively related to voice behavior (β = 0.10, *p* < 0.05), H1 was supported.

**TABLE 4:** Regression results of emotional exhaustion and self-efficacy.

Variable	Emotional exhaustion						Self-efficacy					
	Model 1		Model 2		Model 3		Model 4		Model 5		Model 6	
	β	S*E*	β	S*E*	β	S*E*	β	S*E*	β	S*E*	β	S*E*
Intercept	5.95***	1.03	5.23***	1.01	5.88***	1.00	4.39***	0.72	4.10***	0.72	4.45***	0.71
***Controls***												
Gender	0.07	0.11	0.12	0.10	0.14	0.10	−0.11	0.07	−0.09	0.07	−0.08	0.07
Age	−0.63*	0.26	−0.64*	0.25	−0.61*	0.25	−0.06	0.18	−0.06	0.18	−0.09	0.18
Education	−0.10	0.09	−0.16	0.09	−0.16	0.09	0.04	0.07	0.02	0.07	0.03	0.07
Tenure	0.08*	0.03	0.08*	0.03	0.07*	0.03	−0.01	0.02	−0.01	0.02	−0.01	0.02
***Independent variable***												
POQ			0.22***	0.06	0.14*	0.07			0.09*	0.04	0.11**	0.04
***Moderator***												
Careerism orientation					0.25**	0.07					−0.07	0.06
***Two-way interaction***												
POQ × CO					0.11	0.06					0.11*	0.05
*R* ^2^	0.02		0.06		0.08		0.01		0.02		0.05	
Δ*R*^2^	–		0.04		0.06		–		0.01		0.04	

*N* = 430. Age and tenure were log-transformed using natural logarithms. Statistics reported are unstandardized regression coefficients, standard errors (SEs). POQ, perceived overqualification, CO, careerism orientation. *p < 0.05, **p < 0.01, ***p < 0.001.

**TABLE 5:** Regression results of voice behavior.

Variable	Voice behavior					
	Model 7		Model 8		Model 9	
	β	S*E*	β	S*E*	β	S*E*
Intercept	3.09***	0.76	2.76***	0.75	2.05**	0.75
***Controls***						
Gender	−0.10	0.08	−0.07	0.08	−0.03	0.08
Age	0.18	0.18	0.18	0.18	0.13	0.17
Education	0.12	0.07	0.10	0.08	0.08	0.07
Tenure	0.00	0.02	0.00	0.02	0.01	0.02
***Independent variable***						
Perceived overqualification			0.10*	0.05	0.09*	0.05
***Mediator***						
Emotional exhaustion					−0.10*	0.04
Self-efficacy					0.30***	0.07
*R* ^2^	0.01		0.02		0.12	
Δ*R*^2^	—		0.01		0.11	

*N* = 430. Age and tenure were log-transformed using natural logarithms. Statistics reported are unstandardized regression coefficients, standard errors (SEs). *p < 0.05, **p < 0.01, ***p < 0.001.

Next, section “Results” showed that POQ was positively related to emotional exhaustion (β = 0.22, *p* < 0.001), and emotional exhaustion was negatively related to voice behavior (β = −0.10, *p* < 0.05). Moreover, POQ was positively related to self-efficacy (β = 0.09, *p* < 0.05), and self-efficacy was positively related to voice behavior (β = 0.30, *p* < 0.001). In order to test the mediation effect of emotional exhaustion and self-efficacy, the bootstrapping results of the indirect effects analyses were shown in Table 6. According to the results, the indirect effect of emotional exhaustion between POQ and voice behavior was significant (*indirect effect* = −0.023, 95% CI [−0.051, −0.005]), indicating that H2 was fully supported. The indirect effect of self-efficacy between POQ and voice behavior was significant (*indirect effect* = 0.027, 95% CI [0.005, 0.058]), indicating that H3 was fully supported.

**TABLE 6:** The bootstrapping test for mediation effect.

Path	Effect value	SE	Boot 95% CI	
			Lower	Upper
Path 1: POQ → EE → VB	−0.023	0.011	−0.051	−0.005
Path 2: POQ → SE → VB	0.027	0.014	0.005	0.058
Diff indirect effect (path 1–path 2)	−0.049	0.016	−0.086	−0.022

*N* = 430. SE, standard errors; CI, confidence interval; POQ, perceived overqualification; EE, emotional exhaustion; SE, self-efficacy; VB, voice behavior.

In addition, according to the results in Table 4, the moderating effect of careerism orientation on the relationship between POQ and emotional exhaustion was non-significant (β = 0.11, *p* = 0.065). Thus, Hypothesis 4a was not supported.

**FIGURE 2:**
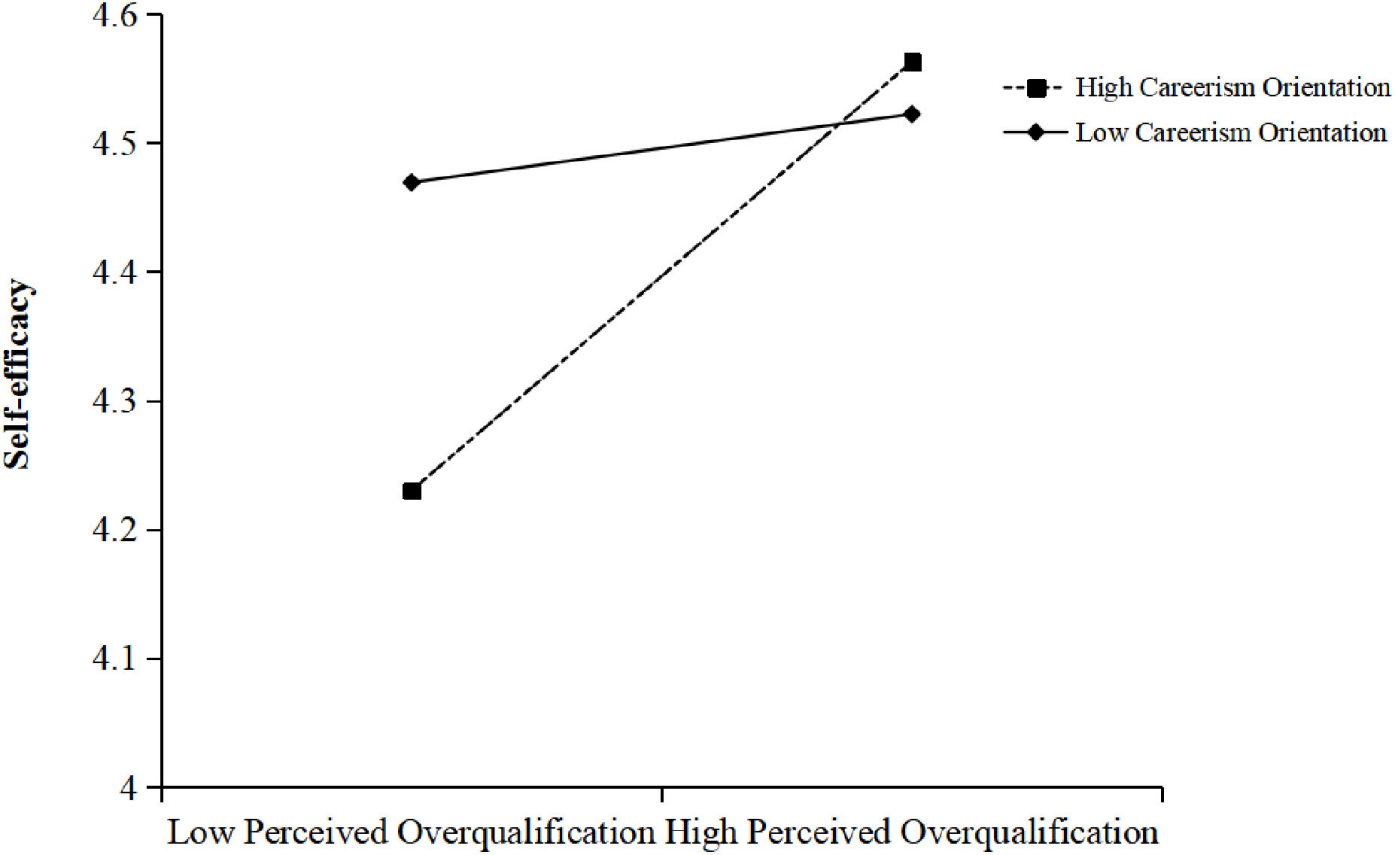
Simple slope of careerism orientation.

Similarly, the moderating effect of careerism orientation on the relationship between POQ and self-efficacy was significant (β = 0.11, *p* < 0.05). The simple slope test in Figure 2 shown that, when careerism orientation was high (*M* + 1 SD) (*simple slope* = 0.19, *p* < 0.01), the relationship between POQ and self-efficacy was significant. While when careerism orientation was low (M − 1 SD) (*simple slope* = 0.03, *p* = 0.59), the relationship was not significant. These results support Hypothesis 4b.

In order to test the moderated mediation effect, we followed Edwards and Lambert’s (2007) procedure to calculate the conditional indirect effects at 1 SD above and below the mean of moderator. As shown in Table 7, when careerism orientation was high (M + 1 SD), the indirect effect of POQ on voice behavior via emotional exhaustion was significant (*indirect effect* = −0.023, 95% CI [−0.058, −0.004]). While the indirect effect was non-significant (*indirect effect* = −0.006, 95% CI [−0.030, 0.009]) when careerism orientation was low (*M* − 1 SD). However, the two cases the difference was significant (*indirect effect* = −0.018, 95% CI [−0.051, −0.001]), supporting Hypothesis 5a.

**TABLE 7:** The bootstrapping test for moderated mediation effect.

Path	Effect value	SE	Boot 95% CI	
			Lower	Upper
**Perceived overqualification→ emotional exhaustion→ voice behavior**				
High careerism orientation (*M* + SD)	−0.023	0.013	−0.058	−0.004
Low careerism orientation (*M* − SD)	−0.006	0.009	−0.030	0.009
Difference between high and low	−0.018	0.012	−0.051	−0.001
**Perceived overqualification→ self-efficacy→ voice behavior**				
High careerism orientation (*M* + SD)	0.059	0.022	0.022	0.108
Low careerism orientation (*M* − SD)	0.009	0.018	−0.026	0.044
Difference between high and low	0.050	0.027	0.010	0.116

*N* = 430; CI, confidence interval

Similarly, the indirect effect of POQ on voice behavior via self-efficacy was significant (*indirect effect* = 0.059, 95% CI [0.022, 0.108]) when careerism orientation was high (*M* + 1 SD). While the indirect effect was non-significant (*indirect effect* = 0.009, 95% CI [−0.026, 0.044]) when careerism orientation was low (*M* − 1 SD). However, the two cases the difference was significant (*indirect effect* = 0.050, 95% CI [0.010, 0.116]), supporting Hypothesis 5b.

## 5 Conclusion and contribution

### 5.1 Conclusion

This article, based on the JD-R model, presents a double-edged sword mechanism by which POQ influences employees’ voice behavior, supported by empirical study involving data from 459 employees. The study concludes with the following findings: POQ is positively related to employees’ voice behavior; the effect of POQ on employees’ voice behavior exhibits a double-edged sword effect under different mediating mechanisms. Specifically, emotional exhaustion partially mediates the relationship between POQ and employees’ voice behavior, where POQ positively influences emotional exhaustion, which in turn negatively affects voice behavior. Conversely, self-efficacy also partially mediates this relationship, as POQ positively impacts employees’ self-efficacy, which may contribute to greater engagement in voice behavior. When comparing the two mediating pathways, the mediating effect of self-efficacy (the positive effect pathway) is stronger than that of emotional exhaustion (the negative effect pathway). In this context, self-efficacy represents a key personal resource that enables employees with POQ to better cope with work challenges, thus enhancing their willingness and capacity to engage in voice behavior. Emotional exhaustion, on the other hand, reflects a depleting job demand that negatively affects voice behavior but exerts a comparatively weaker mediating effect. This suggests that POQ employees prioritize leveraging their personal resources, such as self-efficacy, to overcome work stressors rather than being hindered by emotional exhaustion.

### 5.2 Theoretical contribution

First, this study introduces a double-edged sword perspective to understand the impact of POQ on employee voice behavior. Prior research has reported inconsistent findings—some highlighting the disengaging and suppressive effects of POQ (e.g., Yang and Li, 2021; Wu et al., 2017), while others emphasize its motivating potential via interactions with person-organization (P-O) fit (e.g., Erdogan et al., 2018). However, few studies have systematically integrated these opposing effects within a unified explanatory framework. Addressing this gap, the present study adopts the JD-R model to conceptualize POQ as a dual-faceted job perception that can simultaneously hinder and facilitate employee voice behavior through distinct emotional and motivational processes.

Second, drawing on the dual-pathway proposition of the JD-R model (Demerouti et al., 2001; Schaufeli and Bakker, 2004), this study develops a parallel mediation model that identifies emotional exhaustion and self-efficacy as key mechanisms linking POQ to voice. While prior studies have loosely associated POQ with various negative emotional states such as anger (Liu et al., 2015), boredom (Kim et al., 2019), job burnout (Sánchez-Cardona et al., 2020), contempt, and jealousy, they often lack theoretical integration. This study refines existing research by positioning emotional exhaustion as a core outcome along the health-impairment pathway and self-efficacy as a core resource in the motivational pathway, thereby offering a theoretically grounded account of how POQ can concurrently deplete and energize employees.

Third, this study introduces careerism orientation as a critical individual difference variable that moderates the relationship between POQ and employee outcomes. While existing research on the boundary conditions of POQ has primarily emphasized external situational or cultural factors (e.g., institutional context and labor market structure; Simon et al., 2019; Wang et al., 2019; Zheng and Wang, 2017), this study highlights how internalized career values—particularly instrumental motives for advancement—shape employees’ responses to overqualification. In doing so, it extends the JD-R model by incorporating a value-based individual moderator, offering a more nuanced understanding of when and for whom POQ becomes a source of motivation or strain.

### 5.3 Practical contribution

First, this study provides insights for managers on effectively encouraging POQ employees to engage in voice behavior. Previously, managers often held a negative view of POQ, perceiving such individuals as “arrogant” and mismatched with their roles, which in turn increased burnout (Sánchez-Cardona et al., 2020) and turnover intentions (Lobene et al., 2015; Ye et al., 2017; Wu and Chi, 2020), ultimately incurring human resource costs. However, people are multifaceted. This article highlights that the proximal and distal effects of POQ on employee voice behavior can be positive. Employees with POQ are often more capable and confident in tackling complex tasks and challenges (Chen and Zhou, 2025; Hu et al., 2015), which can stimulate beneficial voice behavior and knowledge sharing that contribute to organizational change and progress (Zhou et al., 2020; Erdogan et al., 2018). Although the direct effect of POQ on voice behavior is small, it remains practically important. Voice is a low-frequency but high-impact behavior; even small increases can contribute to meaningful improvements in problem-solving and decision-making. Moreover, voice behaviors accumulate and spread within teams, so small individual effects can grow into significant group-level outcomes. Therefore, managers should recognize and value the positive contributions of POQ employees to the organization (Wu et al., 2022; Vǎtǎmǎnescu et al., 2023).

Second, organizations should focus on creating a psychologically safe environment to proactively prevent the negative impacts of POQ on employees’ voice behavior. The findings indicate that POQ can heighten employees’ feelings of job insecurity and emotional exhaustion. Therefore, managers should enhance their attention to employees’ mental health by providing psychological support and counseling services. This support can help employees manage the negative emotions arising from POQ, enabling them to express their opinions freely even when experiencing such feelings. This, in turn, can enhance their sense of involvement and encourage active participation in voice behavior.

Third, organizations should provide personalized training and development opportunities for employees with POQ to stimulate creative thinking and enhance their proactive engagement at work. This study finds that employees with POQ tend to have higher self-efficacy, which may contribute to greater participation in voice behavior. Therefore, following the resource conservation model (Hobfoll et al., 2018), managers should create an organizational environment that enhances employee efficacy by increasing work challenges and complexity. This approach can help bridge the psychological gap for POQ employees, enabling them to leverage their excess resources for value addition and enhancement through their self-efficacy.

### 5.4 Limitations and future directions

First, although emotional exhaustion and self-efficacy significantly mediated the relationship between POQ and voice behavior, the magnitudes of the indirect effects were relatively small. These modest effects may be attributed to the influence of unmeasured third variables, such as leadership style, organizational climate, or team support, which could moderate employees’ emotional and cognitive responses and thereby weaken the indirect pathways. Second, there may be a ceiling effect associated with voice behavior. In some organizational or cultural contexts, opportunities for employees to express their opinions are structurally or normatively constrained, resulting in a restricted range of the outcome variable. These factors may jointly suppress the observed mediating effects. Future studies could explore these potential influences by incorporating broader contextual variables and examining their moderating roles in the proposed mechanisms.

Second, the analysis of the moderating effects yielded mixed results, with one hypothesized moderation path reaching statistical significance while the other did not. This suggests that the measurement and operationalization of relevant variables, as well as data collection procedures, may benefit from further refinement. Future research should explore boundary conditions related to individual goals and careerism orientation in greater depth, including identifying and applying appropriate theoretical frameworks to better explain these moderating effects. Additionally, subsequent studies could investigate how careerism orientation differentially moderates various dimensions of POQ, thereby enriching understanding of its complex role.

Third, this study measures POQ from the employees’ perspective, which may lead to inflated relationships between variables due to self-assessment. Furthermore, the current data and research methods (such as multiple time-point data collection) do not support strong causal inferences, as the research is retrospective and lacks a longitudinal design, leaving the possibility of reverse causation. Future studies could consider longitudinal designs and experience sampling methods, as well as supervisor ratings or objective indicators (e.g., education-job mismatch) to mitigate issues of subjectivity and measurement bias.

Fourth, there is the issue of the generalizability of the research. Since the sample in this article consists of employees from the financial industry in China, it does not take into account the generalizability across different industries and regions. Additionally, factors such as cultural differences, management styles, and individual traits introduce variability, meaning the conclusions may not hold in broader or more diverse contexts. Furthermore, the scales used in this study were developed based on Western organizational environments. Future research should consider local scales that reflect Chinese characteristics and culture, expanding the understanding of POQ within the Chinese context. This includes exploring implicit and hard-to-measure qualifications, such as relational resources in Chinese society, to enhance the reliability and consistency of the results.

Finally, the initial sample comprised 800 participants, of whom 430 were included in the final analysis, resulting in an attrition rate of approximately 46.25%. Due to confidentiality constraints and the nature of third-party data collection, only fully matched and validated datasets were accessible, precluding any comparison between retained and dropped participants. This inability to examine differences raises the possibility of attrition bias, which may compromise the external validity and generalizability of the findings. Future research should prioritize strategies to retain data on all participants or implement longitudinal designs to better understand the characteristics and implications of sample attrition.

## Data Availability

The raw data supporting the conclusions of this article will be made available by the authors, without undue reservation.
